# A Cross‐Disease Microglial Transcriptional Program Characterizes Neurodegeneration and Highlights SPP1 as a Biomarker

**DOI:** 10.1002/glia.70163

**Published:** 2026-04-21

**Authors:** Alessandro Palma, Roberta Stefanelli, Francesco Trenta, Chiara Projetti, Greta Massa, Sonia Canterini, Maria Teresa Fiorenza

**Affiliations:** ^1^ Division of Neuroscience, Department of Psychology Sapienza University of Rome Rome Italy; ^2^ PhD Program in Behavioral Neuroscience Sapienza University of Rome Rome Italy; ^3^ European Center for Brain Research, IRCCS Fondazione Santa Lucia Rome Italy

**Keywords:** genomics, macrophages, microglia, neurodegeneration, Niemann‐Pick, SPP1

## Abstract

Microglial cells are key players in maintaining brain homeostasis and responding to pathological conditions. Their multifaceted roles in health and disease have garnered significant attention in the context of neurodegeneration. In recent years, single‐cell transcriptomic techniques have provided unprecedented insights into microglial heterogeneity, revealing distinct subpopulations and gene expression patterns associated with neuroprotection or neurotoxicity. Here, we dissect the transcriptomic landscape of microglia by leveraging human single‐nuclei RNA sequencing datasets from multiple neurodegenerative conditions, including Amyotrophic Lateral Sclerosis, frontotemporal dementia, Alzheimer's disease, aging, and Parkinson's disease. This integrative analysis identifies distinct microglial subpopulations, reflecting functional heterogeneity across diseases and reveals a shared cross‐disease microglial transcriptional program associated with inflammatory and neurodegenerative processes. Using a machine learning framework, we further demonstrate that this transcriptional program enables robust discrimination between neurodegenerative and control samples. Experimental validation in primary microglia isolated from a mouse model of Niemann‐Pick disease type C, also known as juvenile Alzheimer's disease, supports the conservation of key components of this program and highlights Spp1 as a biomarker of disease‐associated microglia states. Overall, this study provides an improved portrait of microglia transcriptional remodeling across neurodegenerative disorders and offers a framework for identifying conserved molecular features that may inform therapeutic strategies aimed at modulating microglial activity to mitigate disease progression and foster neuroprotection.

## Graphical abstract

Distinct microglial subpopulations are depicted, highlighting variations in gene expression and functional roles in response to specific disease conditions and the complexity of microglial activation and differentiation in neurodegeneration.

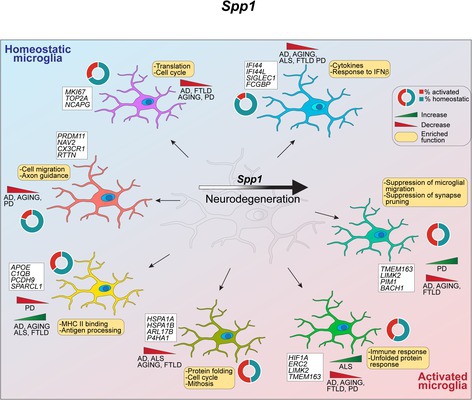

**Abbreviations**
AD: Alzheimer's disease
ALS: amyotrophic lateral sclerosis
CNS: central nervous system
FTLD: frontotemporal lobar dementia
PD: Parkinson's disease

## 1 Introduction

Microglia are the myeloid cells of the central nervous system (CNS), and play a crucial role in the developing brain, maintaining brain homeostasis, and responding to various pathological conditions (Li and Barres 2018; Hanisch and Kettenmann 2007; Wolf et al. 2017). These specialized phagocytes are now recognized as multifaceted actors in both health and disease, exerting numerous functions vital to the brain's proper functioning. This includes shaping the extracellular matrix surrounding synapses (Crapser et al. 2021), maintaining and regenerating the myelin sheath (Kent and Miron 2024; Lloyd and Miron 2019), and establishing crosstalk with other cells (Ronzano et al. 2021; Touil et al. 2023), among others.

In the context of neurodegenerative disorders, such as Alzheimer's disease (AD), Parkinson's disease (PD), amyotrophic lateral sclerosis (ALS), aging‐related cognitive decline, and frontotemporal lobar dementia (FTLD), microglia have garnered significant attention due to their diverse functions and dynamic responses (Salter and Stevens 2017; Hickman et al. 2018; Gao et al. 2023). It is widely recognized that in AD, microglia are implicated in the clearance of amyloid‐beta plaques (Hu et al. 2023), one of the disease's hallmarks. However, dysfunctional microglia can contribute to neuroinflammation, exacerbating neuronal damage and cognitive decline (Leng and Edison 2021). In PD, the role of microglia in disease pathogenesis remains under investigation, but their participation in neuroinflammation is increasingly recognized as a critical contributor to disease progression. Microglial activation, triggered by misfolded alpha‐synuclein aggregates, leads to the release of pro‐inflammatory cytokines and neurotoxic factors, contributing to dopaminergic neuron degeneration (Hickman et al. 2018; Joers et al. 2017). In ALS, microglia play a double role, exerting both neuroprotective and neurotoxic effects. Indeed, while microglia initially attempt to clear misfolded proteins and damaged neurons, chronic activation can lead to the release of cytotoxic molecules, exacerbating motor neuron degeneration (Vahsen et al. 2021). Furthermore, in the context of neurodegeneration, including aging, microglia undergo phenotypic changes termed “microglial priming” (Perry and Holmes 2014; Niraula et al. 2017). Primed microglia exhibit exaggerated inflammatory responses to stimuli and impaired resolution of inflammation, thus contributing to cognitive decline and increased vulnerability to neurodegenerative diseases. Finally, microglia‐mediated neuroinflammation is a prominent feature of FTLD, which is defined by the specific degeneration of frontal and temporal lobes. Dysregulated microglial activation can lead to cytokine release, oxidative damage, and mitochondrial impairment, thus contributing to synaptic dysfunction and neuronal loss in affected brain regions (Bright et al. 2019).

Understanding the complex roles of microglia in neurodegenerative disorders is essential for developing targeted therapeutic interventions aimed at modulating microglial function to mitigate disease progression and promote neuroprotection in affected individuals. Moreover, studies that go beyond the dualistic classification of microglia (Rosa Paolicelli et al. 2022) are needed for a more comprehensive understanding of microglia in physiology and disease.

In recent years, the advent of single‐cell transcriptomic techniques has revolutionized our understanding of cellular heterogeneity and dynamics. These cutting‐edge methodologies have enabled researchers to dissect the transcriptomes of individual cells, providing unprecedented insights into the diversity and functional states of various cell types, including brain‐resident cells. For instance, by profiling thousands of individual cells, studies have revealed previously unrecognized subpopulations within the microglia (Masuda et al. 2020), each exhibiting distinct gene expression patterns and functional properties. By deciphering the transcriptional signatures associated with neuroprotective or neurotoxic microglial phenotypes, it could be possible to uncover potential therapeutic targets for modulating microglial responses in neurodegenerative disorders.

Here, an integration of distinct human single‐nuclei RNA sequencing (snRNAseq) datasets of AD, AGING, ALS, FTLD, and PD patients, together with their cognate healthy controls, has been performed to dissect the transcriptomic landscape of microglia in both health and disease. The integrated analysis revealed important transcriptional programs and regulatory networks linked to inflammation, neurodegeneration, and aberrant cell behavior. Genes belonging to the disease‐associated transcriptional program have been validated on a bulk transcriptomic dataset of neurodegenerative microglia and by immunofluorescence and real‐time quantitative PCR in a mouse model of Niemann‐Pick type C (NPC) disease.

Despite being classified as a lysosomal storage disease with aberrant lipid and cholesterol metabolism, NPC is characterized by neuroinflammation (Cologna et al. 2015) and pathological hallmarks (Gujjala et al. 2026) similar to those of the major neurodegenerative disorders. Specifically, increased microglial and T‐lymphocyte activation, along with the expression of chemotaxis pathway genes and IFN‐γ‐ and IFN‐α‐responsive genes, occur in the pre‐symptomatic stages of NPC disease (Shin et al. 2019).

In our overall findings, we identify Spp1 as a promising marker of microglia in neurodegeneration.

## 2 Materials and Methods

### 2.1 Single‐Cell RNA Sequencing Data Processing

Publicly available single‐nuclei RNA sequencing data have been downloaded from the Gene Expression Omnibus (GEO) repository under the following accession numbers: GSE243292, GSE219281, GSE174332. Parkinson's disease data have been downloaded from https://doi.org/10.5281/zenodo.7886802 (Dehestani et al. 2024). Analyses have been conducted with the *Seurat v5* package (Hao et al. 2024) in the R environment. Each dataset has been separately pre‐processed, selecting only the microglial cell population whenever the dataset also contained other cell types. For datasets lacking predefined microglial annotations (e.g., GSE219281), cell identities were reassigned based on cluster‐specific marker enrichment. Clusters expressing canonical microglial/myeloid markers were selected, and expression of established microglial identity genes was verified after integration. After merging the datasets, a further subset refinement has been performed to exclude potential perivascular macrophage populations.

Data have been normalized, scaled, and the top 2000 variable features computed and used for downstream analyses. Principal component analysis (PCA) has been run, and the first 30 principal components (PCs) have been used to find neighbors and clusters at a 0.3 resolution. Finally, the UMAP algorithm has been run with the first 30 PCs, using the “*umap‐learn*” algorithm. Following this initial merging step, batch effects across datasets were addressed using Harmony‐based integration. Specifically, the merged Seurat object was harmonized using the Harmony integration method, after which layers were joined, and the standard Seurat workflow was applied to re‐cluster cells and perform downstream analyses using the Harmony embedding as the default reduction.

### 2.2 Cell Population Classification and Markers

To find markers for each cluster, the “*FindAllMarkers*” function in *Seurat* has been used, with a minimum percentage of expression of 25% and a log_2_ fold change threshold of 0.25, retrieving both positive and negative markers. Gene ontology analysis on population markers has been performed using the “*ClusterProfiler*” (Wu et al. 2021) R package, utilizing all ontologies (GO BP, GO CC, and GO MF), and 10,000 permutations. Results from the functional enrichment analysis, together with the top expressed markers (positive markers) for each cell subpopulation, have been used to assign a functional profile to each microglial cell subpopulation. A cluster expressing ependymal cell markers (Cluster 4) was identified and excluded prior to downstream analyses to restrict subsequent analyses to bona fide microglia.

### 2.3 Microglial Transcriptional Program Scoring Analysis

To evaluate the distribution of microglial transcriptional programs across conditions, a curated gene set approach was employed. Gene lists were manually curated from the literature and included well‐established markers associated with homeostatic microglial identity (*P2RY12, TMEM119, CX3CR1, TREM2, SALL1, OLFM3, CSF1R, FCRLS, MEF2C, GPR34*), and genes commonly upregulated in damage‐associated or reactive microglial states (*APOE, SPP1, CST7, LPL, CD9, ITGAX, CHI3L1, AIF1, GPNMB, TREM2*). *TREM2* appears in both lists because it participates in state transitions and exhibits context‐dependent expression dynamics. The “*AddModuleScore*” function of the *Seurat* package was then used to compute scores for each cell. Density plots were drawn using a *ggplot2* customized function and were edited with *Adobe Illustrator 2025*.

### 2.4 Pseudobulk Analysis

Pseudobulk differential expression analyses were performed by aggregating single‐nucleus counts at the patient level. For disease versus control comparisons, raw counts were aggregated per patient and condition using in‐house scripts, and differential expression was computed using DESeq2 (Love et al. 2014). All control (non‐pathological) samples were pooled and used as a reference for each disease comparison.

For the homeostatic versus immune‐related microglia analysis, cells were aggregated per patient and microglial state using the *AggregateExpression* function in Seurat. Differential expression was then performed using the *FindMarkers* function with *test.use = “DESeq2*”. In both analyses, genes with adjusted *p*‐value < 0.05 were considered significantly differentially expressed unless otherwise specified.

Enrichment analysis on GO terms (GO biological processes, cell components, and molecular functions) associated with differentially expressed genes for each pathology was performed with *EnrichR* (Kuleshov et al. 2016) in the R environment, using an adjusted *p*‐value threshold of 0.05 for significant GO terms and plotting only the top 10 significant terms based on the adjusted *p*‐value ranking of GO terms. Heatmaps of gene signatures were drawn using the “*pheatmap*” (Kolde 2019) package. Volcano plots were drawn using the “*EnhancedVolcano*” package. All images were edited with *Adobe Illustrator 2025*.

### 2.5 Random Forest Model for Disease Status Classification

To assess whether microglial transcriptional profiles could discriminate between disease conditions, a random forest classifier was trained using normalized single‐cell expression data extracted from the Seurat object. Cells were randomly split into training (70%) and test (30%) sets, stratified by disease condition.

Feature selection was performed exclusively on the training set to avoid data leakage. Specifically, genes were ranked by variance across training cells, and the top 150 most variable genes were retained as input features for model training.

The random forest model was implemented using the random forest R package, with disease condition (CTRL, AGING, AD, ALS, FTLD, PD) as class labels. Model performance was evaluated on the held‐out test set using confusion matrices and receiver operating characteristic (ROC) curves. Area under the curve (AUC) values were calculated for each class using the pROC package.

Feature importance was assessed based on mean decrease in accuracy, and the top‐ranking genes were visualized. Correct and incorrect predictions were projected onto the Harmony‐integrated UMAP embedding using Seurat metadata and custom R scripts.

The image of the confusion matrix was edited using *GraphPad Prism v7*, and the UMAP images were refined with *Adobe Illustrator 2025*.

### 2.6 Animal Models and Microglia Isolation

*Npc1* ^*−/−*^ mice with BALB/CJ background, obtained from heterozygous crosses, were exposed to a 12 h light–dark cycle and received food and water *ad libitum*. Pup genotypes were identified by PCR analysis of tail DNA. Sex‐ and age‐matched littermates were group‐housed in standard cages (Size: 13 cm height × 26 cm length × 20 cm width) enriched with a transparent red polycarbonate igloo house.

Experimental protocols were approved by the Italian Ministry of Health, and experiments were conducted according to the ethical and safety rules and guidelines for the use of animals in biomedical research provided by the relevant Italian laws and the European Union's directives (Italian Legislative Decree 26/2014 and 2010/63/EU). Wild‐type (*wt*) littermates were used as controls in all experiments.

Whole brains from *wt* and *Npc1* ^*−/−*^ young adult mice (postnatal day 30 and 60, named PN30 and PN60, respectively) were dissected and enzymatically dissociated into a single‐cell suspension using the Adult Brain Tissue Dissociation Kits (Cat. Nos. 130‐092‐628 and 130‐107‐677, Miltenyi Biotec) according to the manufacturer's instructions. For each sample, two brains were collected and pulled.

Microglia were positively selected by incubating the cell suspension with CD11b‐conjugated microbeads (Cat. No. 130‐093‐636, Miltenyi Biotec), followed by magnetic separation using MS columns (Cat. No. 130‐042‐201, Miltenyi Biotec). The purity of the isolated microglial population was confirmed by immunofluorescence with Cx3cr1 antibody, which demonstrated the nearly complete purity of our preparations (mean purity = 93.3%) (Figure S3). The isolated CD11b‐positive microglia were either immediately processed for RNA extraction or cultured in DMEM/F12 medium supplemented with 10% heat‐inactivated fetal bovine serum (FBS), 5 ng/mL TGF‐β1, and penicillin/streptomycin solution at 37°C in a humidified atmosphere with 5% CO_2_ for 3 days. Cultured cells were subsequently used for immunofluorescence analysis.

### 2.7 Immunofluorescence of Cells and Cryosections

Freshly isolated microglia from *wt and Npc1* ^*−/−*^ mice were fixed in 4% paraformaldehyde (PFA) for 10 min at room temperature, followed by three washes with phosphate‐buffered saline (PBS). Cells were then permeabilized in PBS containing 0.1% Triton X‐100 for 10 min. After permeabilization, blocking was performed using 0.3% bovine serum albumin (BSA) in PBS for 30 min at room temperature.

Cells were incubated for 1 h at room temperature with primary antibodies against Cx3cr1 (Elabscience, Cat. No. E‐AB‐34231) and Spp1 (Santa Cruz Biotechnology, Cat. No. sc‐21742), diluted in blocking solution. Following three washes with PBS, cells were incubated with the appropriate secondary antibodies: goat anti‐mouse Alexa Fluor 488 for Cx3cr1 (Invitrogen, ref. A11034) and goat anti‐rabbit Alexa Fluor 555 for Spp1 (Invitrogen, ref. A32727), for 1 h at room temperature in the dark. Finally, cells were washed three times in PBS and counterstained with Hoechst 33258 (Sigma‐Aldrich, 1:5000) before imaging.

Twenty‐micrometer‐thick cryosections from *wt* and *Npc1* ^*−/−*^ mice were fixed in 4% PFA for 10 min at room temperature, followed by three washes with PBS. Cells were then permeabilized in PBS containing 0.3% Triton X‐100 for 15 min. After permeabilization, blocking was performed using 5% horse serum, 0.5% BSA, 0.05% Tween‐20 in PBS for 1 h at room temperature. Cryosections were incubated overnight at 4°C with primary antibodies against Iba1 (GeneTex, Cat. No. GTX638147) and Spp1 (Proteintech, Cat. No. 22952‐1‐AP), diluted in blocking solution. Following three washes with PBS, cryosections were incubated with the appropriate secondary antibodies: goat anti‐mouse Alexa Fluor 555 for Iba1 (Invitrogen, ref. A32727) and goat anti‐rabbit Alexa Fluor 488 for Spp1 (Invitrogen, ref. A11034), for 1 h at room temperature in the dark. Finally, sections were washed three times in PBS and counterstained with Hoechst 33258 (Sigma‐Aldrich, 1:5000) before imaging at the fluorescence microscope.

### 2.8 RNA Extraction and Real‐Time Quantitative PCR

RNA was extracted with the Total RNA Purification kit (Norgen, Cat. 48400) following the manufacturer's instructions. One microgram RNA was reverse transcribed into cDNA using Maxima H Minus Reverse Transcriptase (Thermo Scientific, EP0752). Primers used for the real‐time quantitative PCR (RT‐qPCR):

Hprt1 forward: CGTGATTAGCGATGATGAACCA.

Hprt1 reverse: CACACAGAGGGCCACAATGT.

Spp1 forward: TCTCCTTGCGCCACAGAATG.

Spp1 reverse: TGTGGTCATGGCTTTCATTGGA.

RT‐qPCR was performed in three biological replicates with three technical replicates, using Hprt1 as a housekeeping gene. Gene expression was analyzed using the 2^−ΔΔCt^ method. Statistical analysis was conducted on the ΔCt values (assuming a normal distribution), applying a *t*‐test to determine significant differences in relative expression.

### 2.9 RNA Sequencing Analysis

Raw counts were downloaded from the GEO database (GSE109083). The count matrix was given as input to DESeq2 (Love et al. 2014) in the R environment and processed following a standard pipeline. Differentially expressed genes were taken as genes with adjusted *p*‐value < 0.05. Data were imported into GraphPad v7 (Prism) and edited with Adobe Illustrator 2025.

### 2.10 Statistical Analysis and Figures

Statistical values in charts are reported as mean ± SEM unless otherwise specified. The statistical significance is defined as Bonferroni adjusted *p*‐value where *p < 0.05, **p < 0.01, ***p < 0.001, ****p < 0.0001. For each type of analysis, see the specific Section 2 and figure captions. Figures were prepared and edited using *Adobe Illustrator 2025* and *GraphPad* (*Prism*) v7 software.

## 3 Results

### 3.1 Functional Heterogeneity of the Microglial Cell Populations

**FIGURE 1:**
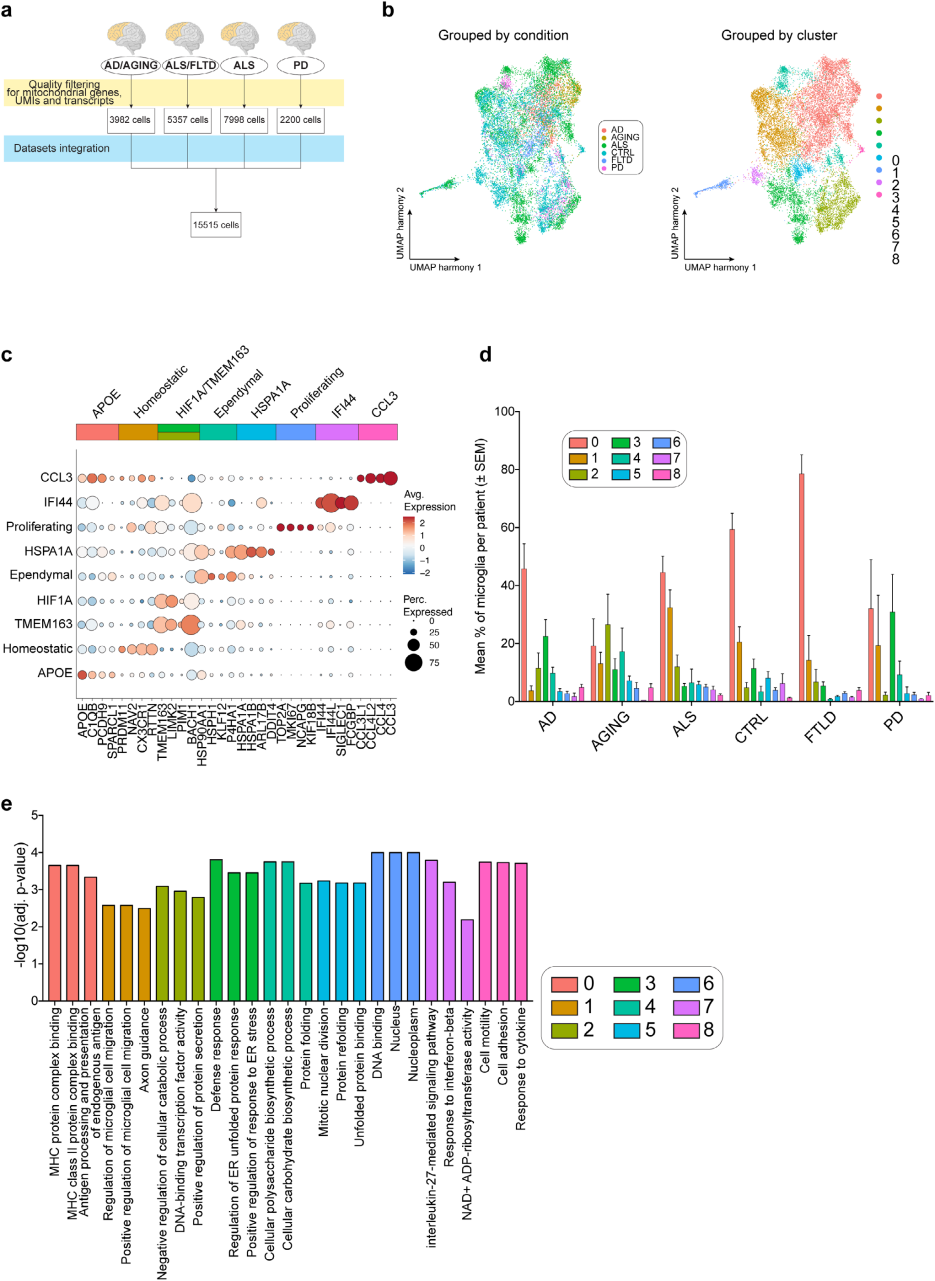
Single‐cell analysis of microglia in healthy and neurodegenerative conditions. (a) Sample stratification chart showing the number of cells per dataset before and after quality control filtering. (b) UMAP projection of the integrated dataset illustrating the distribution of cells by condition (left) and by microglial cluster identity (right). (c) Dot plot displaying the top four marker genes for each microglial subpopulation, indicating both the percentage of expressing cells and the average expression level. (d) Mean proportion of microglial clusters per patient across conditions (±SEM). Cluster proportions were calculated for each individual and averaged within disease groups, revealing enrichment of Cluster 0 in AD, AGING, and FTLD while preserving multiple microglial populations across all conditions. Cluster colors correspond to those shown in the UMAP and dot plot (Panels b and c). (e) Gene set enrichment analysis highlighting the most significantly enriched and activated pathways (adjusted *p*‐value < 0.01).

Publicly available single‐nuclei RNA sequencing data were retrieved from the Gene Expression Omnibus (GEO) database. The datasets comprised post‐mortem, prefrontal cortex samples from individuals with ALS, AD, PD, FTLD, AGING, and their non‐pathological counterparts (Figure 1a). After merging the datasets, correcting batch effects via harmony integration (Figure S1) and applying quality filtering, 15,515 cells were retained and underwent downstream processing. Clustering analysis revealed the existence of nine sub‐populations (Figure 1b), indicative of the functional heterogeneity of microglial cells.

The differentially expressed genes (DEGs) specifically marking each cell cluster (Table S1), which are markers of each cell subpopulation, were used to study the functional heterogeneity of microglial cells. In particular, markers exhibiting the highest positive values (among the top 10 genes ordered in decreasing log_2_ fold change) were used to retrieve information about the functional identity of the individual sub‐populations (Figure 1c). While several markers were found to be expressed by different clusters to varying degrees, an overarching pattern emerged, revealing a distinctive signature for certain subpopulations. Cluster 0 showed increased expression of *APOE* together with complement and antigen presentation genes, including *C1QB*, *HLA‐DRA*, and *CD74*, consistent with an immune‐engaged microglial transcriptional state. When cluster proportions were calculated per patient and averaged across conditions, Cluster 0 was enriched in AD, AGING, and FTLD (Figure 1d). Importantly, despite this enrichment, multiple microglial populations were preserved across all conditions, indicating condition‐associated shifts in microglial states rather than complete replacement of homeostatic microglia. Functional enrichment analysis further revealed that genes upregulated in Cluster 0 were significantly associated with major histocompatibility complex class II (MHC II) binding and antigen presentation pathways, including *HLA‐DRA*, *HLA‐DRB1*, *CD74*, and *CD81* (Figure 1e), supporting enhanced immune‐related activity within this microglial subset.

Cluster 1 displayed elevated levels of *CX3CR1*, *PRMD11*, and *NAV2*, alongside other markers indicative of a quiescent or homeostatic‐like phenotype. The enrichment of terms related to migration and axon guidance of the genes expressed by this population may reflect the developmental or maintenance role of microglia in shaping neural circuits. Importantly, this population displays low expression of disease‐associated or activation markers, reinforcing the idea that it may represent a surveying microglia. Additionally, its marked reduction in terms of abundance was observed in AD and AGING (Figure 1d).

Cluster 2 exhibits elevated levels of *TMEM163*, *LIMK2*, *PIM1*, and *BACH1*, with a gene signature similar to Cluster 3, yet characterized by distinct additional markers. Cluster 2 abundance was markedly decreased in AD, AGING, and FTLD and increased in PD. This microglial subpopulation shows enrichment for gene ontology terms such as “suppression of microglial migration” and “suppression of synapse pruning,” indicating a functionally restrained or hypoactive state. The expression of *TMEM163* and *BACH1* further supports a transcriptional program marked by limited motility, reduced synaptic engagement, and altered regulatory function.

Conversely, Cluster 3 expresses higher levels of *HIF1A* compared to Cluster 2, and its biomarkers enrich terms related to the immune response and unfolded protein response (UPR). Together, these features define Cluster 3 as a metabolically stressed and immune‐reactive microglial population, distinct from the more inhibited Cluster 2. While Cluster 2 appears to reflect functional suppression, Cluster 3 may be in a transitioning or compensatory state, responding to environmental stress through HIF1A‐driven and UPR‐linked mechanisms, potentially balancing immune function and cellular survival.

Cluster 4 displays a transcriptional profile distinct from canonical microglial populations. It is characterized by high expression of genes involved in protein folding and metabolic regulation, including *HSPH1*, *HSP90AA1*, *PTGES3*, *GAB1*, and *P4HA1*. Gene ontology analysis reveals enrichment for pathways related to carbohydrate and glycogen biosynthesis, alongside downregulation of immune cell activation, further suggesting a non‐canonical microglia identity. The presence of transcription factors such as *KLF12* and *ZBTB16*, along with epithelial‐ and neurodevelopment‐associated transcripts like *RNASET2*, *ZFPM2‐AS1*, and *CACNA1A*, suggests that this cluster likely represents a population of ependymal or choroid plexus–derived cells. Given the absence of key microglial markers and their divergent functional signature, Cluster 4 was excluded from downstream analyses focused on microglial diversity and activation.

Cluster 5 is defined by high expression of stress‐inducible heat shock proteins, particularly *HSPA1A* and *HSPA1B*, which are key components of the protein‐folding machinery under conditions of cellular stress. Functional enrichment analysis reveals activation of pathways related to protein folding and cell cycle/mitosis, along with suppression of lipid biosynthesis, suggesting that these cells are in a transiently activated state associated with proteotoxic stress and proliferation. Additional expressed genes such as *DDIT4*, *DUSP1*, *NIBAN1*, and *FOXP1* support a phenotype associated with stress adaptation, transcriptional reprogramming, and regulation of apoptosis. Despite this apparent protective or responsive state, this population is notably reduced in abundance in neurodegenerative conditions, including AD, AGING, ALS, and FTLD. This decline suggests a possible loss of a homeostatic or stress‐buffering microglial state, which may render the brain more vulnerable to disease‐related damage. The presence of *IFNAR2*, *PLP1*, and *BCL6* also hints at potential crosstalk between interferon signaling, glial interaction, and transcriptional regulation in maintaining this cell population.

Consistent with other reports, a cluster of proliferating cells was identified (Cluster 6), displaying high expression levels of *MKI67* and other cell cycle‐related markers (*TOP2A*, *NCAPG*, *KIF18B*, *POLQ*, among others), with reduced abundance observed in all pathological conditions.

Finally, Clusters 7 and 8 represent pro‐inflammatory microglial subpopulations distinguished by their transcriptional response to immune stimuli and cytokines. Interestingly, Cluster 7 exhibits a transcriptional program characterized by elevated expression of type I interferon‐responsive genes, including *IFI44*, *MX1*, and *STAT1*, along with *SIGLEC1* and *CD163L1*, suggesting a microglial state responsive to cytokine signaling. However, despite this apparent pro‐inflammatory signature, the cluster itself was predominantly observed in control (CTRL) samples. This apparent discrepancy may reflect the fact that interferon‐responsive microglia do not necessarily represent a damage‐associated activated state, but rather a primed state within the spectrum of physiological microglial surveillance. In contrast, Cluster 8 displays strong expression of inflammatory chemokines, including *CCL2*, *CCL3*, *CCL3L1*, *CCL4*, and *CCL4L2*, as well as immune regulators such as *IL1B*, *CD83*, *EGR1*, *HLA‐DRA*, and *CD74*. This transcriptional profile suggests an activated microglial state with enhanced chemotactic capacity. GO enrichment reveals upregulation of cell adhesion, cytokine responses, and inflammation, while endoplasmic reticulum stress response (ERAD) and proteasome pathways are suppressed, implying a trade‐off between immune activation and proteostatic control. While Cluster 8 shares some features with general activated microglia, the unique upregulation of chemokines suggests a specialized role in immune cell recruitment and neuroinflammatory amplification. Together, Clusters 7 and 8 may represent functionally divergent arms of microglial activation in response to distinct pathological cues.

Collectively, these findings underscore the functional heterogeneity of microglia, which comprise transcriptionally distinct subpopulations with potentially divergent roles in brain homeostasis and disease. The observed shifts in the abundance of specific microglial states across neurodegenerative conditions may reflect not only consequences of pathology but also active contributors to disease progression, highlighting their dual roles as both responders and potential drivers of neurodegeneration.

### 3.2 Projection of Microglial Heterogeneity Onto Homeostatic and Immune‐Related Transcriptional Axes

Microglia display substantial transcriptional plasticity across physiological and pathological conditions, and increasing evidence supports the existence of multiple functional states rather than discrete polarization categories (Rosa Paolicelli et al. 2022; Sankowski and Prinz 2025). To complement our cluster‐based analysis, we projected individual microglial transcriptomes onto two conserved gene expression programs associated with homeostatic identity and immune‐related activation.

Homeostatic and immune‐related gene signatures were constructed based on curated literature, including canonical homeostatic markers (*P2RY12, TMEM119, CX3CR1, TREM2, SALL1, OLFM3, CSF1R, FCRLS, MEF2C, GPR34*) and genes associated with immune engagement and disease contexts (*APOE, SPP1, CST7, LPL, CD9, ITGAX, CHI3L1, AIF1, GPNMB, TREM2*). Notably, *TREM2* appears in both lists because it is required for the transition to activated states but is also expressed at lower levels in homeostatic microglia, hence with a context‐dependent role. Single‐cell scores were then calculated for each signature, allowing visualization of microglia along continuous transcriptional axes rather than binary states.

**FIGURE 2:**
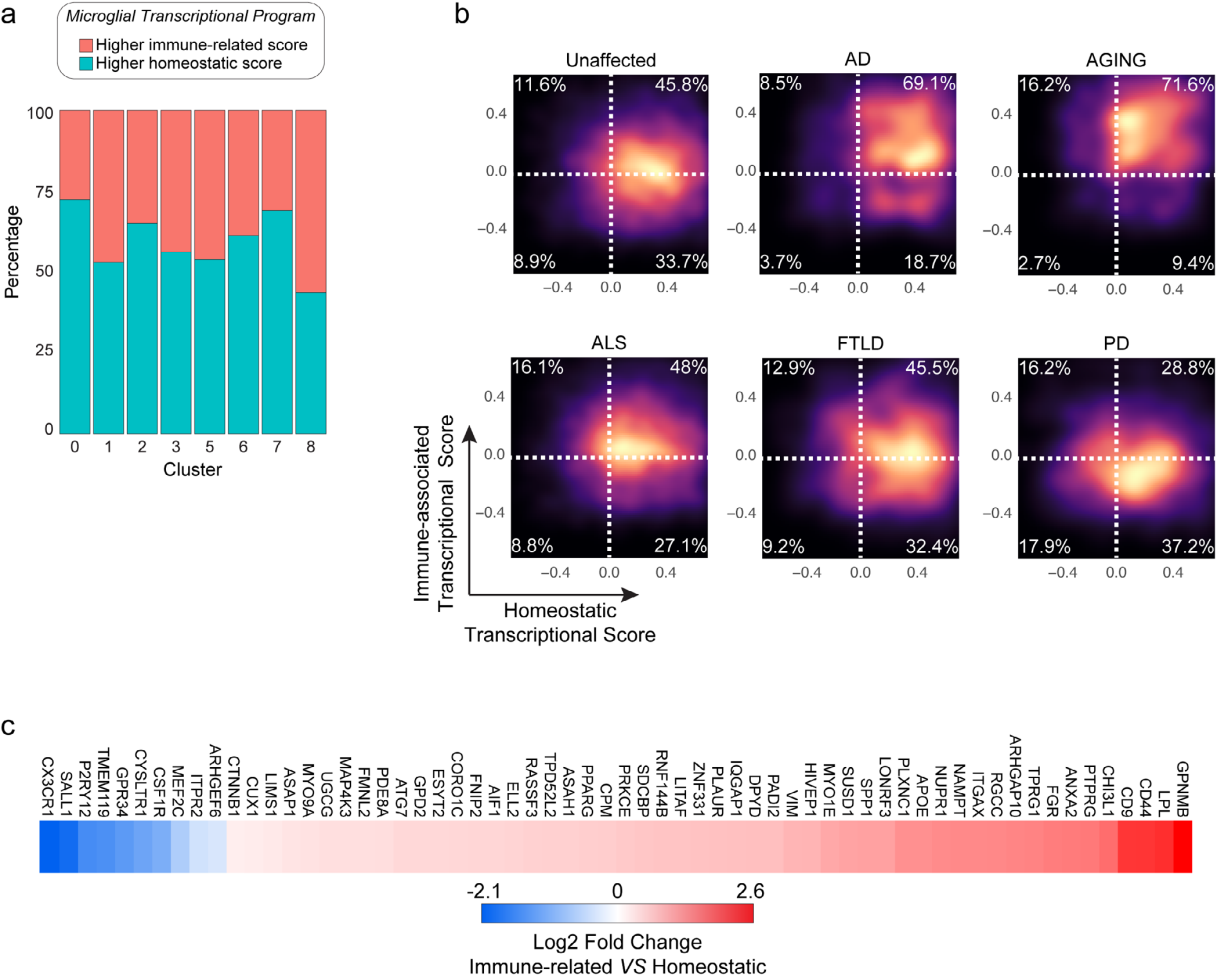
Projection of microglial heterogeneity onto homeostatic and immune‐related transcriptional programs. (a) Stacked bar plot showing the proportion of cells with higher homeostatic or immune‐related gene scores within each microglial cluster. Scores were calculated at the single‐cell level using the AddModuleScore function. (b) Density plots illustrating the distribution of individual microglia along homeostatic and immune‐related gene score axes across conditions. Percentages indicate the fraction of cells within each quadrant. (c) Heatmap displaying the log_2_ fold change of the most differentially expressed genes (adjusted *p*‐value < 0.05) between cells with higher immune‐related versus higher homeostatic gene scores, visualized using a blue–white–red color scale.

This analysis revealed that microglial clusters previously identified distribute heterogeneously across both axes, indicating that cluster identity does not correspond to fixed homeostatic or immune‐engaged states (Figure 2a,b). Instead, individual microglia occupy a spectrum of transcriptional programs within and across clusters.

When stratified by condition, disease‐associated samples showed a shift toward higher immune‐related scores, particularly in AD and AGING, while retaining substantial representation of cells with preserved homeostatic gene expression (Figure 2b). Together, these results indicate that neurodegenerative conditions are associated with graded shifts in microglial transcriptional programs rather than uniform transitions between discrete states, consistent with the heterogeneous cluster structure already observed.

Overall, these results indicate that microglia distribute along continuous transcriptional gradients rather than occupying fixed homeostatic or immune‐engaged states. While certain subpopulations show relative enrichment for immune‐related gene expression programs, substantial heterogeneity is preserved both within and across clusters, suggesting context‐dependent functional modulation rather than binary state transitions.

To further refine the gene programs associated with these transcriptional axes, we performed differential expression analysis by grouping cells according to their relative immune‐related and homeostatic gene scores. This comparison identified 62 differentially expressed genes (Figure 2c and Table S2), delineating a transcriptional profile associated with higher immune‐related scores. Among the genes enriched in cells with elevated immune‐related scores were *GPNMB*, *LPL*, and *CHI3L1*, previously linked to neurodegenerative contexts and to microglial responses to lipid‐rich and inflammatory environments (Liu et al. 2025; Keren‐Shaul et al. 2017; Mwale et al. 2025). These genes are implicated in phagocytosis, lipid metabolism, immune signaling, and extracellular matrix remodeling. Additionally, upregulation of *NAMPT*, *PADI2*, and *ITGAX* further supports enhanced metabolic and immune‐related activity. Conversely, cells with higher homeostatic gene scores displayed increased expression of canonical microglial identity genes such as *TMEM119*, *P2RY12*, *CX3CR1*, and *SALL1*. Notably, the analysis also highlighted genes such as *SPP1*, *PTPRG*, and *ARHGAP10*, which may contribute to microglial functional modulation in disease contexts. Together, these results reinforce the concept that neurodegenerative conditions are associated with graded transcriptional reprogramming of microglia, involving coordinated modulation of homeostatic and immune‐related gene programs rather than uniform state conversion.

### 3.3 Pseudobulk Analysis and the Most Variable Genes Define a Common Neurodegeneration Gene Signature

**FIGURE 3:**
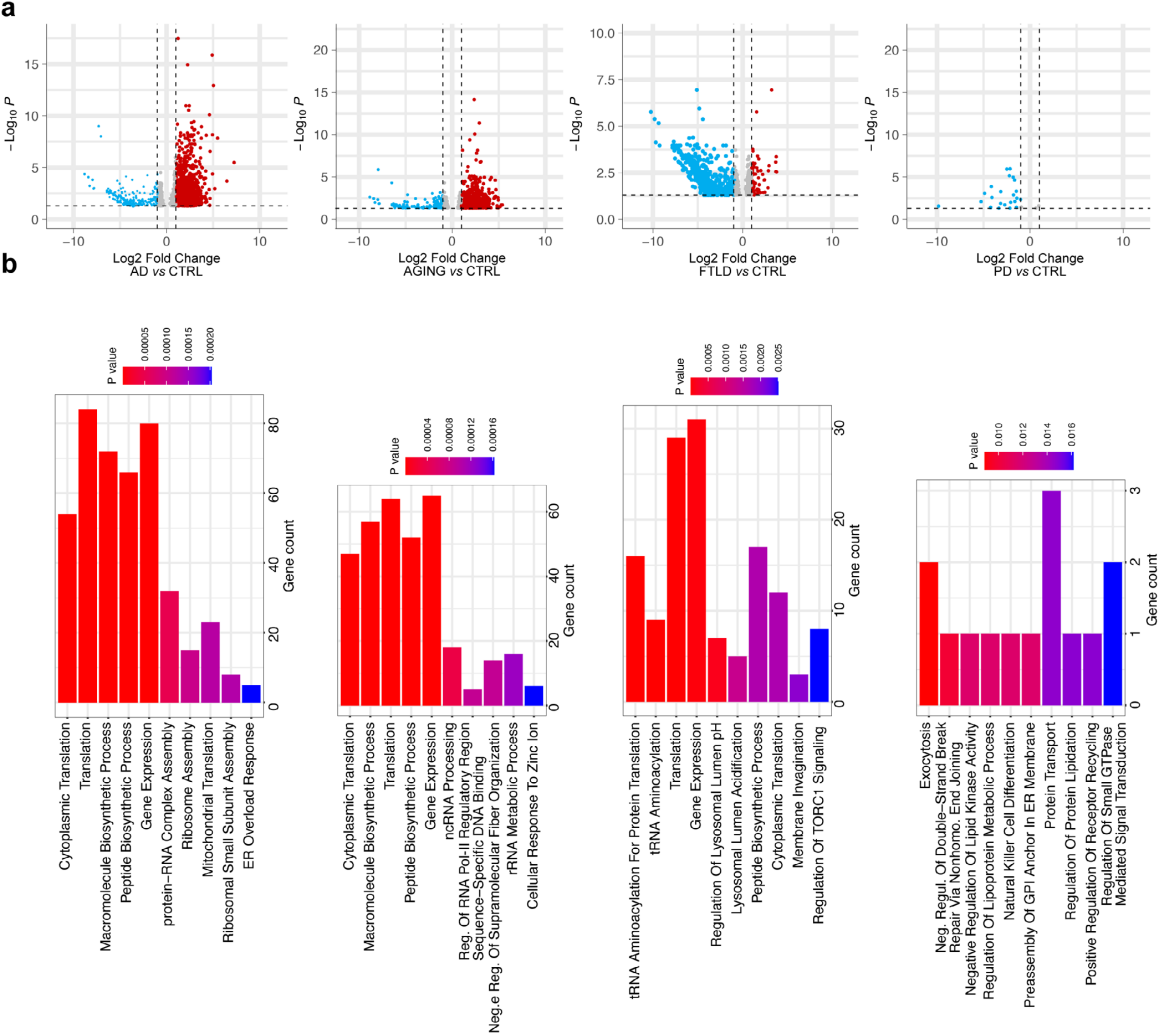
Pseudobulk analysis of microglia in AD, AGING, ALS, and FTLD compared to CTRL. (a) Volcano plots (top panels) displaying differentially expressed genes in each condition versus CTRL. Downregulated genes are shown in blue and upregulated genes in red (adjusted *p*‐value < 0.05). (b) Bar plots (bottom panels) showing the top 10 significantly enriched gene ontology (GO) terms associated with the differentially expressed genes in each condition.

To investigate transcriptional changes associated with neurodegenerative conditions, a pseudobulk differential expression analysis was performed by aggregating microglial transcriptomes per patient and condition. Compared to pooled controls, all disease groups displayed hundreds of significantly up‐ and down‐regulated genes (Figure 3a, Table S3). In contrast, PD did not show robust differentially expressed genes under this cross‐cohort framework. While the original PD study reported substantial microglial differential expression within genetically stratified cohorts and matched controls (Dehestani et al. 2024), our analysis compares PD microglia against a harmonized control population pooled across datasets, which increases stringency and likely reduces detection of mutation‐ or region‐specific effects.

Despite disease‐specific differences, common enrichment patterns emerged across multiple conditions, notably involving cytoplasmic translation, mitochondrial metabolism, and ribosome assembly, particularly in AD and AGING (Figure 3b). In contrast, FTLD showed enrichment in lysosomal pathways and tRNA aminoacylation, while PD was characterized by changes in lipid metabolism (see Table S4 for the complete enrichment analysis).

These results point to a core microglial response to neurodegeneration, centered on cellular stress and protein homeostasis, alongside distinct molecular signatures that may reflect disease‐specific pathological mechanisms.

To characterize transcriptional features shared across neurodegenerative conditions, we intersected the top 2000 highly variable genes from each dataset, identifying 166 genes consistently variable across all conditions (Figure S2). Rather than representing a predictive signature, this gene set defines a shared cross‐disease microglial variability program. Visualization of log_2_ fold changes relative to controls revealed partially concordant transcriptional responses across diseases, with AD and AGING clustering together and FTLD showing substantial overlap, while ALS displayed an intermediate profile and PD appeared more distinct (Figure S2). This shared gene set includes established markers of reactive microglia (*SPP1*, *APOE*, *GPNMB*, *CD163*), regulators of phagocytosis and immune signaling (*MERTK*, *CX3CR1*, *IL1B*), and genes involved in lysosomal and iron metabolism (*FTH1*, *FTL*), supporting the presence of a conserved microglial transcriptional response across neurodegenerative conditions (Table S5).

Importantly, log_2_ fold changes of these genes showed strong concordance across AGING, AD, and FTLD, supporting the presence of conserved transcriptional responses among distinct neurodegenerative contexts (Figure S2).

**FIGURE 4:**
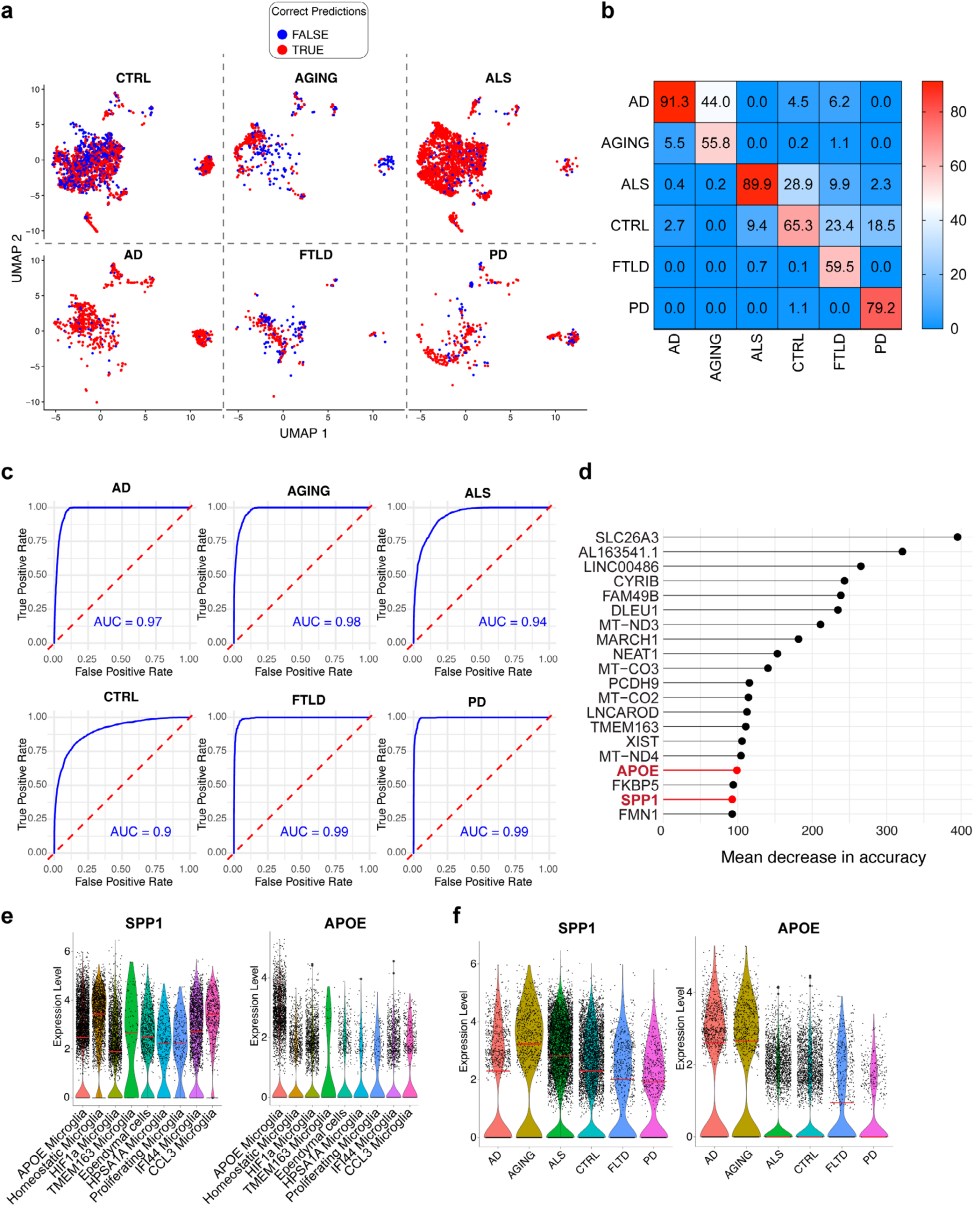
Classification of damage‐associated microglial transcriptional states. (a) UMAP representation of held‐out test microglial cells split by disease condition and colored by prediction accuracy (red, correctly classified; blue, misclassified). (b) Confusion matrix showing the percentage of predicted versus actual disease labels for test cells. (c) Receiver operating characteristic (ROC) curves and corresponding area under the curve (AUC) values (in blue) for each disease condition, calculated on held‐out cells. (d) Top 20 gene features ranked by importance in the random forest model. (e) Violin plots of SPP1 (left) and APOE (right) expression across microglial subpopulations. (f) Violin plots of SPP1 (left) and APOE (right) expression across disease conditions. Median expression values are indicated by red lines.

To assess whether microglial transcriptional profiles could discriminate disease conditions, we trained a random forest classifier using a training/test split framework with feature selection performed exclusively on the training set. Using the top 150 most variable genes from the training data, the classifier achieved an overall accuracy of 77.2% on held‐out cells (*κ* = 0.69), with balanced accuracies ranging from 0.77 to 0.93 across conditions (Figure 4a,b). Consistent with transcriptomic similarities observed across datasets, AD and AGING samples showed partial overlap, whereas PD exhibited high specificity. AUC values ranged from 0.90 to 0.99 across conditions (Figure 4c).

Inspection of the most informative features (Figure 4d) revealed genes involved in mitochondrial function (*MT‐ND3*, *MT‐CO2*, *MT‐CO3*), immune regulation (*CYRIB*, *MARCH1*, *FKBP5*), and cytoskeletal and adhesion pathways (*PCDH9*, *FMN1*), reflecting broad cellular programs associated with microglial activation and stress responses. Notably, *APOE* and *SPP1* emerged among the top‐ranked predictors (top 20), highlighting their relevance within damage‐associated microglial states across independent analytical approaches. While *APOE* is a well‐characterized gene in Alzheimer's disease, and the studies on its role in other neurodegenerative contexts are recently increasing (Jackson et al. 2024; Raulin et al. 2022), less is known about *SPP1*. However, many reports ascribe to SPP1^+^ macrophages a role in both cancerous and non‐cancerous diseases (discussed in Reggio et al. 2025 and reviewed in Palma 2025). Looking at the expression of these genes across the analyzed conditions in the single‐nuclei dataset, *SPP1* appeared to be highly expressed in AGING and ALS, compared to control, while *APOE* was higher in AD, AGING, and FTLD (Figure 4e,f).

Moreover, *SPP1* shows the highest expression in the TMEM163 microglial subpopulation (Cluster 2), whereas *APOE* is predominantly expressed by the immune‐engaged microglial cluster (Cluster 0). These results indicate that these genes could represent potential biomarkers to distinguish damage‐associated from healthy microglia in transcriptomic settings of neurodegenerative disorders.

### 3.4 Spp1 Is Upregulated in the Microglia of a Mouse Model of Neurodegeneration

To experimentally validate the damage‐associated microglial transcriptional program identified in the human single‐nucleus datasets, we employed mice harboring a null mutation of the *Npc1* gene, which recapitulates key pathological features of Niemann‐Pick type C disease, a lysosomal storage disorder characterized by progressive neurodegeneration (Berry‐Kravis 2021). Although this represents a shift from human to murine data, the use of this well‐established model of neuroinflammation‐driven neurodegeneration provides an opportunity to evaluate whether candidate genes associated with microglial activation, and in particular *Spp1*, represent conserved markers of damage‐associated microglial states. Notably, there is a high degree of conservation between the mouse and the human SPP1 protein (Weber 2018).

**FIGURE 5:**
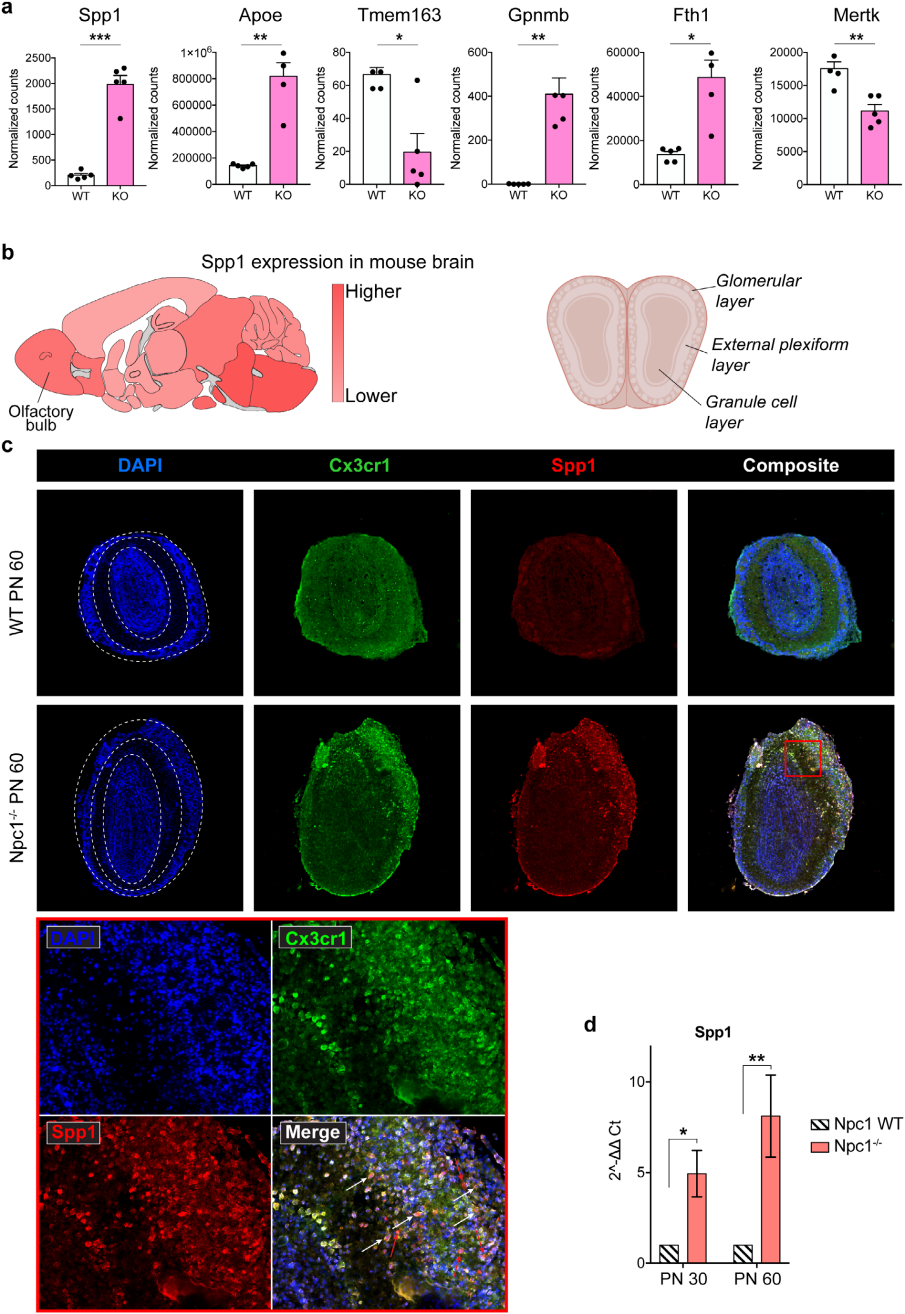
In vivo validation of damage‐associated microglial genes in the *Npc1* ^−/−^ mouse model. (a) RNA‐seq normalized expression levels of selected representative genes (Spp1, Apoe, Tmem163, Gpnmb, Fth1, and Mertk) in wild‐type and *Npc1* ^−/−^ microglia from a publicly available bulk RNA‐sequencing dataset. Genes were selected based on their contribution to the random forest model and/or their inclusion in the shared neurodegenerative transcriptional program. Individual data points represent biological replicates from the neurodegenerative signature. (b) Schematic representation of the mouse brain (adapted from the Human Protein Atlas) displaying average Spp1 expression across distinct brain regions. (c) Immunofluorescence staining of microglia (Cx3cr1), Spp1, and nuclei (DAPI) in representative olfactory bulb sections from WT and *Npc1* ^−/−^ mice. Upper panels show 10× magnification as 3 × 3 combined captures of the entire olfactory bulb; lower panel shows a selected region (corresponding to the red square on the 10× magnification images in the upper panel) at 40× magnification. White arrows point at some of the Spp1‐positive microglia. Red arrows indicate examples of secreted Spp1 localizing close to microglia. (d) Real‐time quantitative PCR (RT‐qPCR) of Spp1 on total mRNA derived from freshly isolated microglia. Log fold change values of WT and *Npc1* ^−/−^ mouse brains at PN30 and PN60 (*n* = 3 per group) are reported. Statistics have been computed on delta Ct for each sample.

We first examined the expression of selected representative genes in a publicly available bulk RNA‐sequencing dataset comparing wild‐type and Npc1‐deficient microglia (Cougnoux et al. 2018). Genes were chosen based on their contribution to the random forest classifier and/or their inclusion in the shared neurodegenerative transcriptional program. This analysis revealed significant dysregulation of multiple genes associated with immune activation and metabolic remodeling, including *Spp1*, *Apoe*, *Tmem163*, *Gpnmb*, *Fth1*, and *Mertk*, in Npc1^−/−^ microglia compared to controls (Figure 5a), supporting the presence of a conserved damage‐associated microglial transcriptional shift in this model.

Next, using the Human Protein Atlas (mouse section), we identified the pons and olfactory bulb as the brain regions with elevated Spp1 expression (Figure 5b). Immunofluorescence assays on olfactory bulb sections from Npc1^−/−^ and *wt* mice confirmed enhanced microglial infiltration in the diseased condition, along with high SPP1 expression. Notably, SPP1 immunoreactivity co‐localized with that of the microglial marker CX3CR1, particularly in the external plexiform and glomerular layers (Figure 5c).

A closer look at Npc1^−/−^ olfactory bulbs revealed that Spp1 is predominantly localized in the cytoplasm within the inner layers (yellow cells, Figure 5c lower panel), whereas in the external plexiform layer it is more frequently detected in a pattern that suggests a secreted form of this protein. At the interface of these two layers, Cx3cr1‐positive microglia with an amoeboid morphology were observed, suggesting a migratory phenotype toward the outer layers of the bulb (Figure 5c lower panel and Figure S4). This spatial pattern may be relevant to the neurodegenerative process, even though the underlying mechanisms remain unclear, and no experimental evidence currently explains this phenomenon.

The determination of *Spp1* transcript levels by real‐time quantitative PCR assays from Npc1‐deficient and *wt* brains showed that *Spp1* expression was significantly upregulated in the microglia of *Npc1* ^*−/−*^ mice, particularly in the PN60 (Figure 5d). This result corroborates the role of this gene in microglial transcriptional rewiring during neurodegeneration.

Altogether, these findings identify a specific transcriptional program of microglia in neurodegeneration, particularly involving *Spp1*, underscoring its potential as a conserved marker of damage‐associated microglial states across neurodegenerative conditions. Further validation of additional genes in human samples will be essential to confirm the broader applicability and translational relevance of the signature.

## 4 Discussion and Conclusions

Microglia, the resident myeloid cells of the central nervous system, are critical regulators of brain homeostasis and active responders to injury and disease. In neurodegenerative disorders, microglia exhibit enhanced phagocytic activity and other features that may contribute to neuronal damage, neuroinflammation, and neurodegeneration (Hickman et al. 2018; Gao et al. 2023). Given their central role in shaping the neurodegenerative environment, we integrated multiple publicly available human single‐nucleus RNA sequencing datasets spanning different neurodegenerative disorders to comprehensively profile microglial transcriptomic heterogeneity in health and disease.

Nine transcriptionally distinct clusters, each associated with specific molecular programs, were identified in our analysis, demonstrating the tremendous functional and transcriptional diversity among microglial populations. Some clusters exhibited disease‐specific variation in abundance, suggesting that specific microglial states are preferentially expanded or diminished in a pathological context‐dependent manner.

Using a pseudobulk differential expression strategy, we identified a conserved neurodegenerative transcriptional program shared across AD, AGING, ALS, and FTLD, pointing to a core microglial response to neurodegenerative stress. Gene ontology enrichment analysis highlighted alterations in immune activation, lipid metabolism, and mitochondrial function, which are hallmarks of neurodegenerative pathology. Several of the genes we identified are known players in neurodegeneration. For example, *APOE*, a key driver of amyloid plaque deposition in AD, is prominently expressed in reactive microglia (Kaji et al. 2024). *SPP1*, although less well‐characterized, is increasingly recognized as a context‐dependent marker of damage‐associated microglia, with both protective and detrimental functions (Palma 2025). Another example is *MERTK*, a receptor involved in the clearance of α‐synuclein fibrils, underscoring its potential role in synucleinopathies (Dorion et al. 2024).

We used a multivariate random forest classifier, which enables classification based on the collective behavior of genes rather than relying on individual differential expression. These models are capable of capturing complex, non‐linear interactions that are not always evident through univariate analyses. Measurements of RNA expression for the most important genes from this model and for the top variable genes within the analyzed datasets, using an independent bulk RNA‐sequencing dataset of microglia from neurodegenerative brains, confirmed differential expression for the majority of these genes, reinforcing the biological relevance of the model. In vivo and in vitro validation in a murine model of Niemann‐Pick type C disease, which recapitulates key features of neurodegeneration, including microglial activation, mitochondrial and lysosomal dysfunction, lipid accumulation, and progressive neurological decline, revealed that not all genes were significantly dysregulated when assessed by real‐time PCR in isolated microglia. This discrepancy highlights an important limitation: machine learning algorithms identify predictive patterns that do not necessarily correspond to differential expression of each individual gene in every context. Nonetheless, the Niemann‐Pick C mouse model provided a crucial biological validation for the genetic signature. We observed an upregulation of *Spp1* gene in RNA‐seq data from *Npc1* ^*−/−*^ microglia. SPP1 expression localized to disease‐relevant brain regions (e.g., olfactory bulb), and was confirmed by immunofluorescence and qPCR in microglia isolated from affected animals. This finding is consistent with our previous study showing that microglia are activated early in the cerebellum and olfactory bulb of *Npc1* mice, at stages that precede overt neuronal involvement and loss, thus underscoring the importance of studying how microglia shape the neurodegenerative environment (Rava et al. 2022). Moreover, findings of this study not only support the robustness of our computational predictions but also underscore the conservation of neurodegeneration‐associated molecular pathways of microglia across diseases.

Prior reports have similarly highlighted SPP1 as a core component of microglial activation in diverse disease contexts, where it may promote phagocytosis, lipid metabolism, and lysosomal function (reviewed in Palma 2025). This suggests that SPP1+ microglia may represent a conserved, disease‐associated phenotype rather than one confined to a specific disorder.

SPP1‐expressing microglia have previously been described as part of the damage‐associated microglia (DAM) program (Lan et al. 2024), a reactive transcriptional state emerging in response to neurodegenerative cues. While DAMs have been proposed to exert context‐dependent and potentially cytoprotective functions (Deczkowska et al. 2018), accumulating evidence suggests that SPP1+ microglia represent a metabolically active and immune‐engaged population often associated with disease contexts (Dräger et al. 2022). Consistent with this framework, our cross‐disease analysis identifies SPP1 as a conserved component of microglial activation across distinct neurodegenerative conditions. Notably, SPP1 expression has also been reported in perivascular macrophages in Alzheimer's disease, highlighting partial overlap between activated microglia and CNS‐associated macrophage populations (De Schepper et al. 2023). Although our study focused on parenchymal microglia, these observations support the existence of shared transcriptional programs among myeloid cells exposed to damage and neurodegenerative environments, rather than strictly disease‐ or compartment‐specific phenotypes.

It is important to acknowledge that experimental validation was limited to a subset of genes, and further studies are needed to delineate the broader functional relevance of the identified microglial states and transcriptional programs. Future research should aim to validate these states in human post‐mortem brain tissue using spatially resolved and single‐cell approaches. Moreover, functional studies in murine models, such as conditional knockouts and pharmacological interventions, will be essential to establish the functional contribution of candidate gene activation, such as SPP1, in the context of neurodegeneration. Integration of longitudinal datasets and patient‐derived microglial models could also provide critical insight into the temporal dynamics of microglial transitions during disease evolution.

In summary, this study reveals disease‐ and context‐specific microglial responses across neurodegenerative disorders, identifies a conserved transcriptomic signature of neurodegeneration, and underscores the value of integrative and multiscale validation. Our findings provide a foundation for future mechanistic investigations and therapeutic targeting of damage‐associated microglial states.

## Author Contributions

Conceptualization, investigation, project administration, supervision, writing – original draft, writing – review and editing: A.P, M.T.F. Data curation: A.P., R.S. Formal analysis: A.P., R.S. Methodology: A.P., R.S., F.T., C.P., G.M. Validation, visualization: A.P., R.S., F.T., C.P., G.M., S.C., M.T.F. Funding acquisition: A.P., S.C., M.T.F.

## Funding

This work was supported by Sapienza Università di Roma (RM123188F700A7B2, RG1221816B9646F5, B83C2500429005), Fondazione Telethon (GSP20006_Covid050).

## Disclosure

During the preparation of this work, the authors used AI‐assisted technologies only to improve the readability and language of the manuscript. After using these tools/services, the authors reviewed and edited the content as needed and take full responsibility for the content of the published article.

## Conflicts of Interest

The authors declare no conflicts of interest.

## Supporting information


**Figure S1:** Dataset integration. UMAP projections showing the overlap between the different datasets before (left panel) and after (right panel) harmony integration.

**Figure S2:** Cross‐disease microglial transcriptional variability program. (a) Intersection of the top 2000 highly variable genes across all datasets identifies 166 genes shared among neurodegenerative conditions. (b) Heatmap of log_2_ fold changes relative to control samples for the 166 shared genes across AD, AGING, ALS, FTLD, and PD, highlighting partially conserved transcriptional responses, particularly among AD, AGING, and FTLD. (c) Pairwise correlations of log_2_ fold changes for shared genes across disease conditions, demonstrating strong concordance among AD, AGING, and FTLD.

**Figure S3:** Validation of isolated microglia identity. Representative immunofluorescence staining of isolated microglia confirming cell purity. Quantification (right) was performed across 30 randomly selected fields at 20× magnification.

**Figure S4:** Validation of isolated microglia identity. (a) Representative immunofluorescence staining of olfactory bulbs from wt mice showing microglia (Iba1, green) and Spp1 (red) at 40× magnification. (b) Representative immunofluorescence staining of olfactory bulbs from Npc1^−/−^ mice showing microglia infiltration (Iba1, green) and Spp1 expression (red) at 40× magnification.

**Table S1:** Table reporting the computed markers for each cell subpopulation (cluster). *p*‐values and adjusted *p*‐values, log_2_ fold changes, clusters, and percentage of cells expressing each marker (pct.1 for the considered cluster, pct.2 for all the other clusters) are reported.

**Table S2:** Table reporting the statistics for differentially expressed genes (DEGS) for each condition against all control samples, related to the pseudobulk analysis performed on cells aggregated by disease condition and patients. File sheets are separated by condition.

**Table S3:** Table reporting the functional enrichment analysis performed on statistically significant genes resulted from the pseudobulk analysis for each disease condition. The data report enrichment statistics related to the enrichment performed on GO biological processes (GO BP), GO cell component (GO CC) and GO molecular function (GO MF) pulled together for each enrichment analysis (each disease vs. ctrl).

**Table S4:** Table reporting the statistics for differentially expressed genes (DEGS) for each condition against all control samples, related to the pseudobulk analysis performed on cells aggregated by disease condition and harmony clusters. File sheets are separated by condition.

**Table S5:** Table reporting the statistically significant genes resulting from the differential expression analysis performed on microglial cells classified as anti‐inflammatory against microglial cells classified as pro‐inflammatory. *p*‐values and adjusted *p*‐values, log_2_ fold changes, and percentage of cells expressing each gene (pct.1 for the anti‐inflammatory cells, pct.2 for pro‐inflammatory cells) are reported.

## Data Availability

Datasets used for this work are available at Gene Expression Omnibus (GEO) repository with the following accession IDs: GSE243292, GSE219281, GSE174332. Parkinson's disease dataset is available from Zenodo repository at the following link: https://doi.org/10.5281/zenodo.7886802. The bulk RNA sequencing dataset is available in GEO database under the accession ID GSE109083.Code used for this work is available from the corresponding author upon reasonable request.
