# Supplementary figures and images for: A Cross‐Disease Microglial Transcriptional Program Characterizes Neurodegeneration and Highlights SPP1 as a Biomarker
Source: Glia. 2026 Apr 21;74:e70163. doi: 10.1002/glia.70163 (PMC13097963; doi:10.1002/glia.70163)

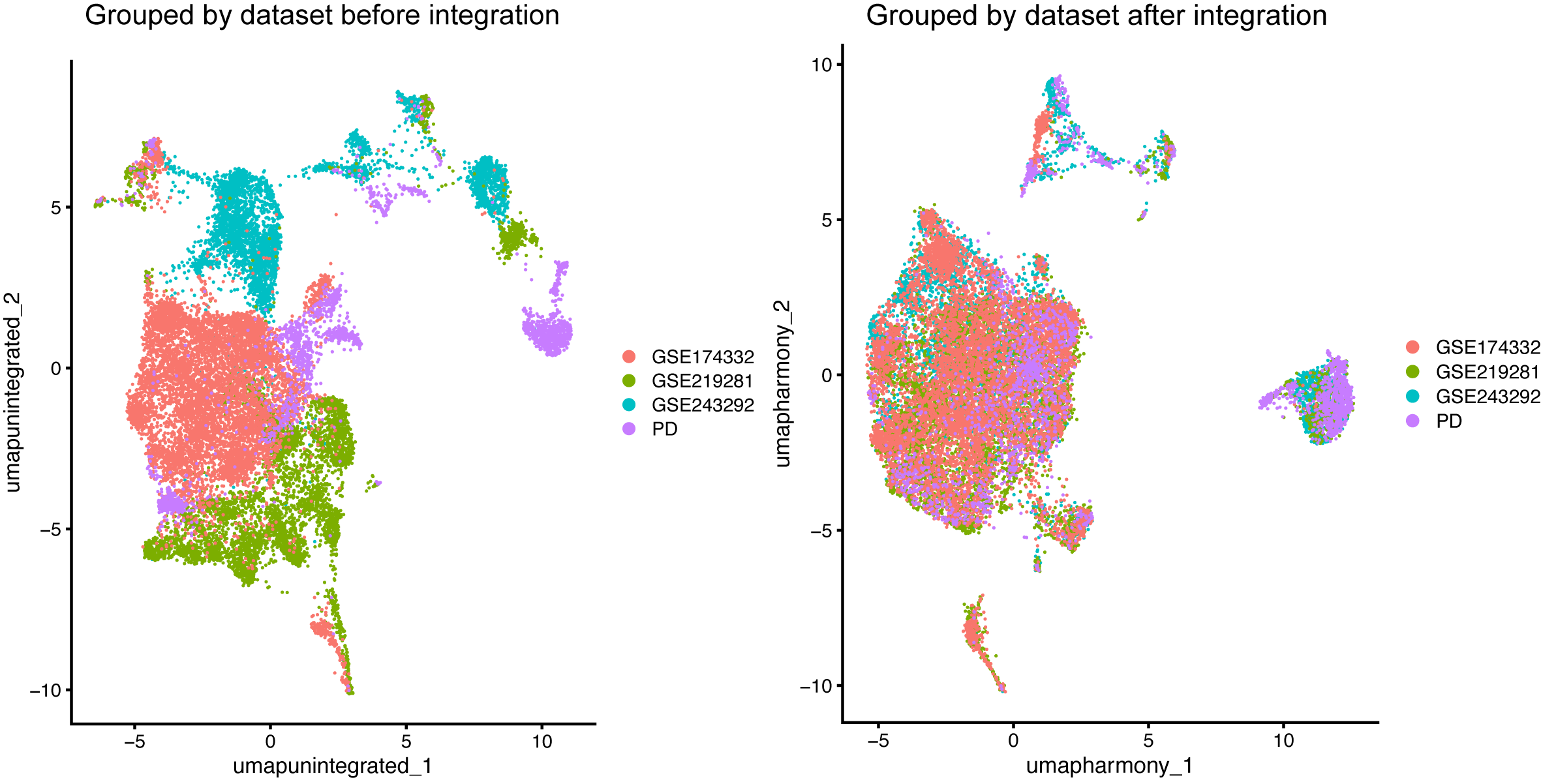

Supplement: Supplementary file 1 — Figure S1: Dataset integration. UMAP projections showing the overlap between the different datasets before (left panel) and after (right panel) harmony integration. [file GLIA-74-0-s007.tif]

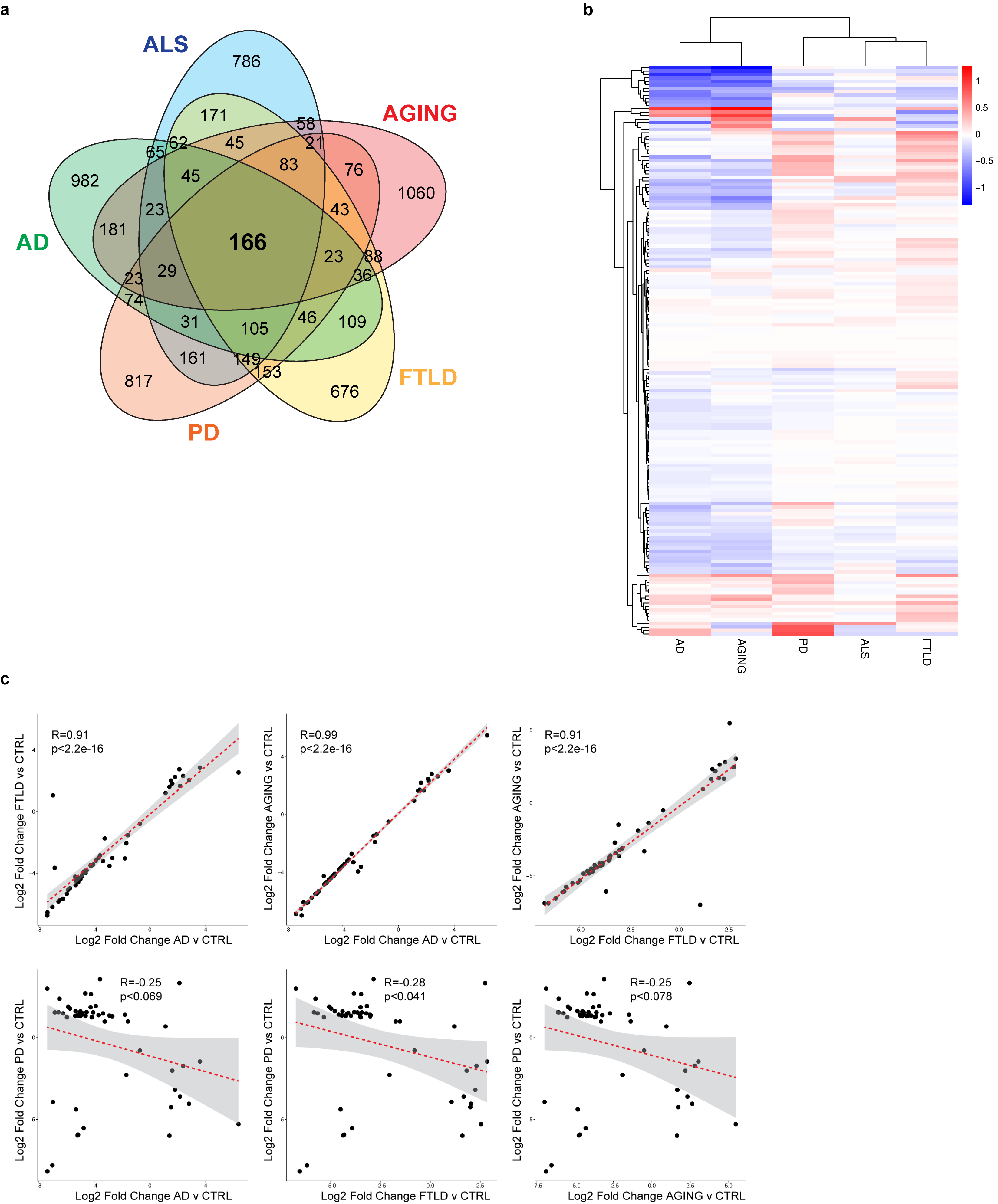

Supplement: Supplementary file 2 — Figure S2: Cross‐disease microglial transcriptional variability program. (a) Intersection of the top 2000 highly variable genes across all datasets identifies 166 genes shared among neurodegenerative conditions. (b) Heatmap of log2 fold changes relative to control samples for the 166 shared genes across AD, AGING, ALS, FTLD, and PD, highlighting partially conserved transcriptional responses, particularly among AD, AGING, and FTLD. (c) Pairwise correlations of log2 fold changes for shared genes across disease conditions, demonstrating strong concordance among AD, AGING, and FTLD. [file GLIA-74-0-s009.tif]

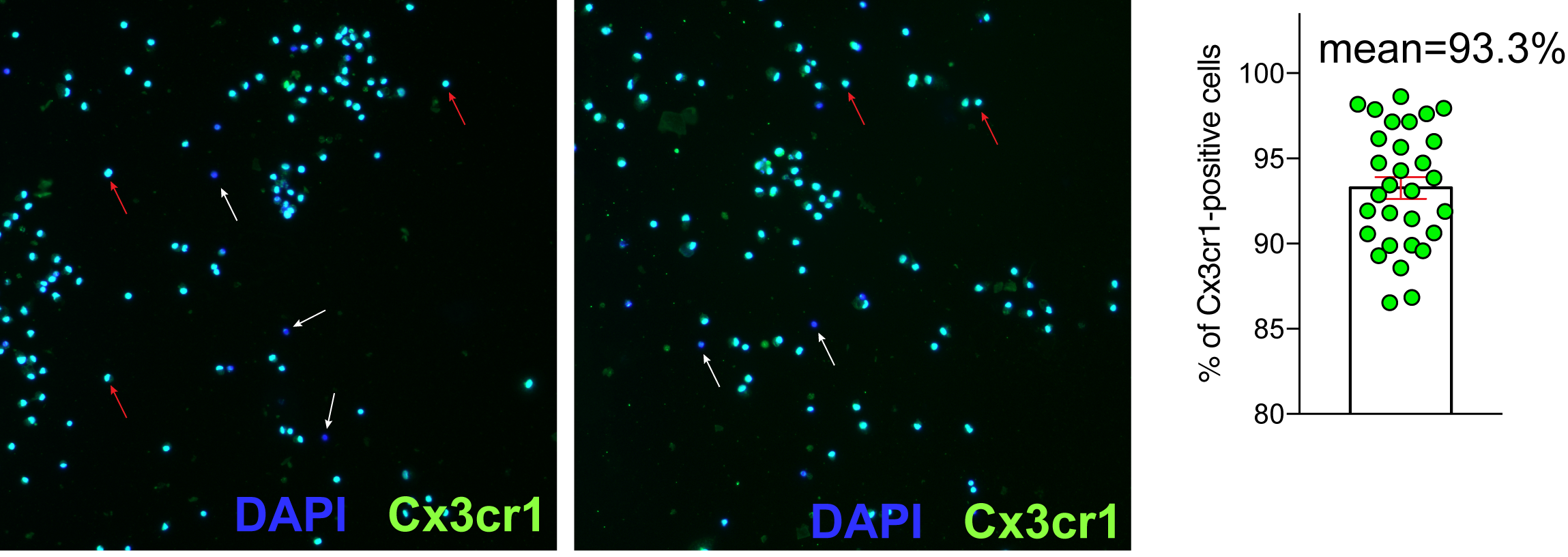

Supplement: Supplementary file 3 — Figure S3: Validation of isolated microglia identity. Representative immunofluorescence staining of isolated microglia confirming cell purity. Quantification (right) was performed across 30 randomly selected fields at 20× magnification. [file GLIA-74-0-s002.tif]

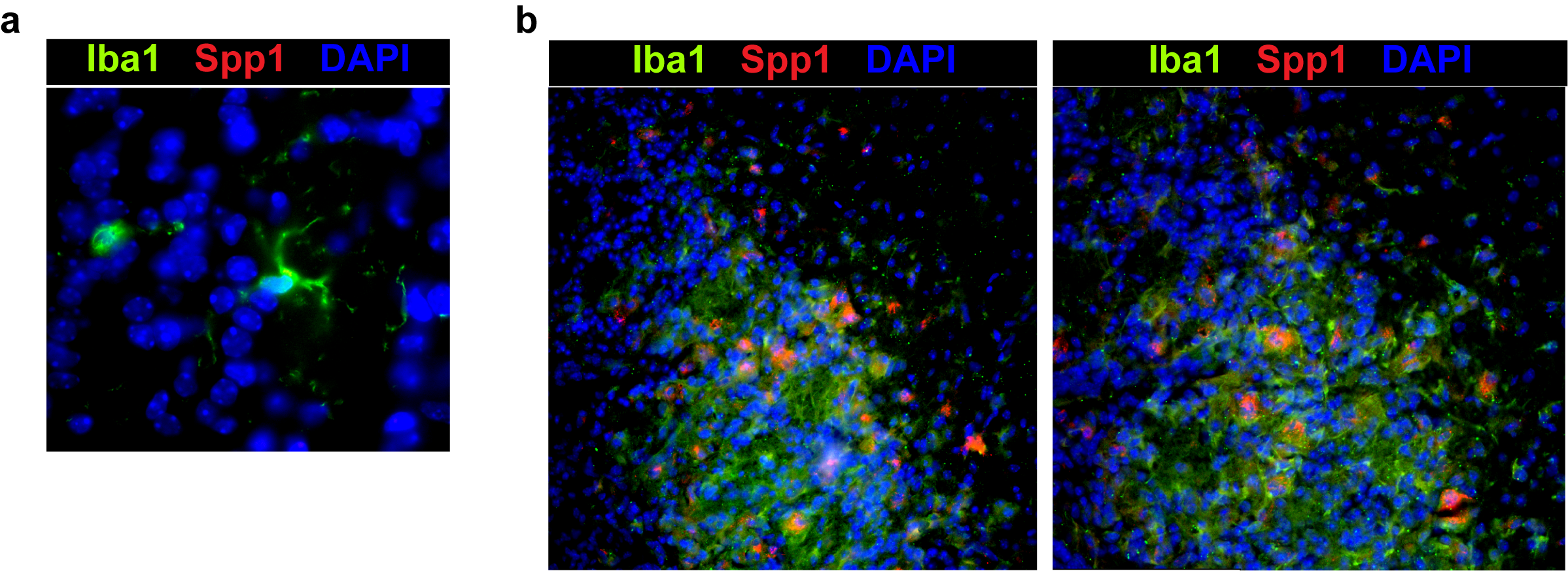

Supplement: Supplementary file 4 — Figure S4: Validation of isolated microglia identity. (a) Representative immunofluorescence staining of olfactory bulbs from wt mice showing microglia (Iba1, green) and Spp1 (red) at 40× magnification. (b) Representative immunofluorescence staining of olfactory bulbs from Npc1−/− mice showing microglia infiltration (Iba1, green) and Spp1 expression (red) at 40× magnification. [file GLIA-74-0-s005.tif]
